# Surgical management of suspected paraneoplastic myasthenia gravis in rectal cancer during neoadjuvant immunotherapy: a case report

**DOI:** 10.3389/fonc.2025.1503362

**Published:** 2025-09-02

**Authors:** Zhiyuan Xia, Haining Chen, Ming Qiu, Wei Huang, Hui Li, Minglin Lin, Sen Zhang

**Affiliations:** Department of Colorectal and Anal Surgery, The First Affiliated Hospital of Guangxi Medical University, Nanning, China

**Keywords:** paraneoplastic myasthenia gravis, paraneoplastic syndrome, immune checkpoint inhibitors, neoadjuvant treatment, locally advanced rectal cancer

## Abstract

Colorectal cancer is among the most common malignancies of the gastrointestinal tract. Immune checkpoint inhibitors (ICIs) have become a key component in the treatment of locally advanced rectal cancer (LARC), offering promising therapeutic outcomes. However, ICIs can occasionally cause significant adverse effects. Herein, we report a case of rectal cancer with suspected paraneoplastic myasthenia gravis (pMG) induced by immune checkpoint inhibitors (ICIs). Unfortunately, the patient lacked anti-acetylcholine receptor (AChR)/muscle-specific kinase (MuSK) antibody testing and muscle biopsy, which precluded a definitive diagnosis of pMG. Remarkably, following surgical resection, the patient not only achieved complete tumor eradication but also experienced full remission of myasthenia gravis (MG).

## Introduction

Colorectal cancer ranks as the third most prevalent malignancy worldwide, with approximately 1.9 million new cases diagnosed annually (1). For patients with locally advanced rectal cancer (LARC), the standard of care includes neoadjuvant chemoradiotherapy followed by total mesorectal excision and adjuvant chemotherapy (2). Recently, immune checkpoint inhibitors (ICIs) have been integrated into the neoadjuvant treatment (NAT) for LARC, yielding promising therapeutic results (3–5). Current evidence on ICIs in LARC predominantly supports their use in microsatellite instability-high (MSI-H) subtypes, where robust responses and guideline-endorsed efficacy have been well documented (5–7). However, since the majority of rectal cancers are microsatellite stable (MSS) (8), and ICIs have not been included as a standard treatment option for MSS rectal cancer in guidelines, ICIs remain investigational for this population. To address this unmet need, our center has initiated a clinical trial (NCT06254521) evaluating neoadjuvant immunotherapy in MSS LARC.

While ICIs have shown significant antitumor efficacy, they are also associated with immune-related neurological complications (9, 10). Myasthenia gravis (MG), an autoimmune neuromuscular disorder primarily caused by autoantibodies targeting acetylcholine receptors at the neuromuscular junction, is commonly observed as a paraneoplastic syndrome (PNS) in patients with thymoma (11), but has also been reported in patients with other malignancies (12–17). However, the occurrence of MG as a PNS in association with rectal cancer has not been reported in the literature.

We present a case of LARC complicated by paraneoplastic myasthenia gravis (pMG), suspected from the symptoms, that developed during NAT with cytotoxic chemotherapy and ICIs. Following curative rectal cancer surgery, the patient achieved complete tumor eradication and full remission of myasthenia gravis.

## Case report

A 55-year-old Chinese man presented to our hospital with a one-year history of altered stool characteristics, including hematochezia. His medical history was notable for chronic hepatitis B, with positive HBsAg and anti - HBc. He had no prior history of myasthenia gravis. Moreover, he had no other comorbidities, such as hypertension, diabetes, or coronary artery disease, and no family history of malignancy or autoimmune diseases.

**Figure 1:**
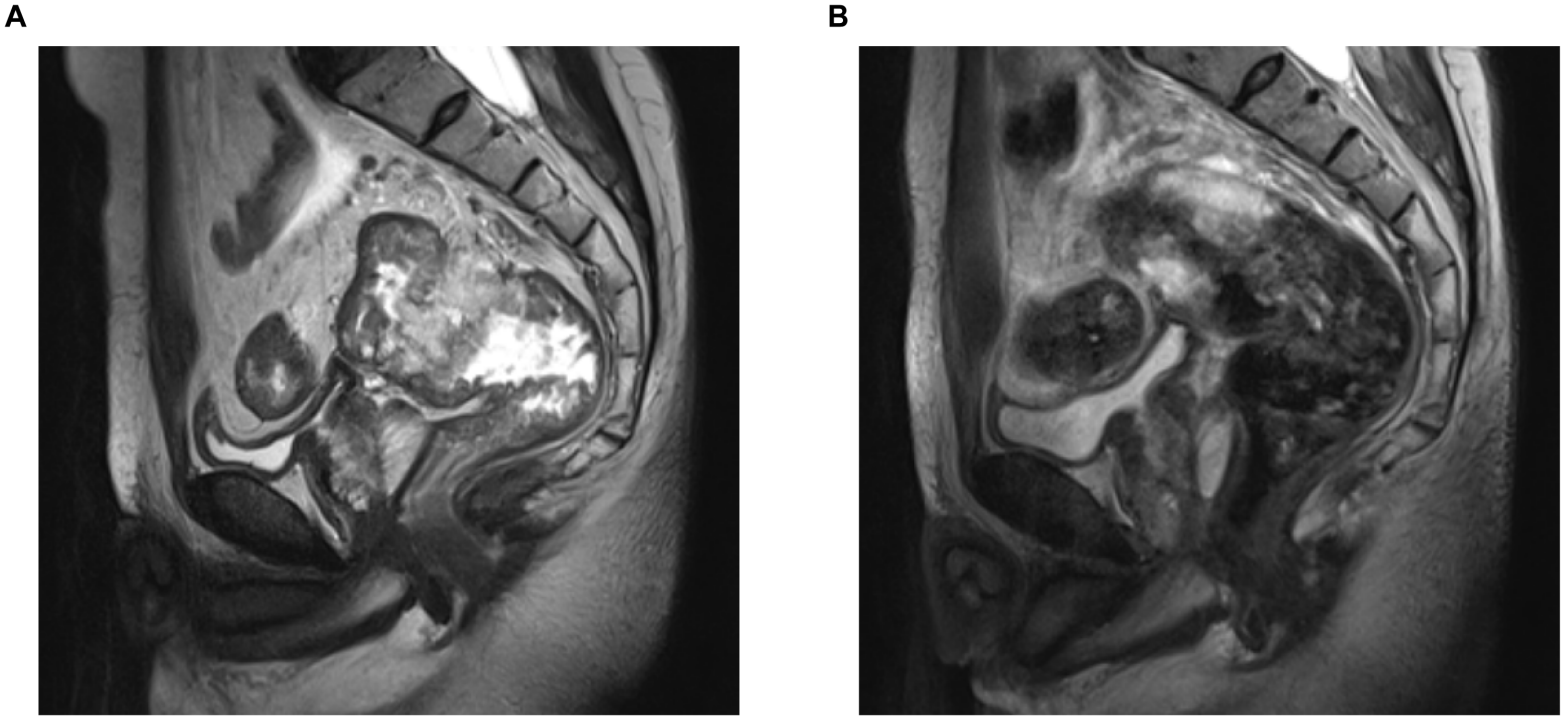
Pelvic MRI of the patient pre- and post-NAT. **(A)** Pre-NAT. **(B)** Post-NAT. NAT, neoadjuvant therapy.

On admission, physical examination revealed a palpable rectal mass, located 7 cm from the anal verge, with limited mobility and fixation on digital rectal examination. Limb muscle strength was intact (grade 5/5), and there was no evidence of eyelid ptosis. Laboratory tests (**Table 1**) revealed elevated tumor markers, with no significant elevation of muscle injury-related enzymes. Pelvic MRI revealed a rectal tumor staged as T4aN2a, with negative mesorectal fascia (MRF-) and no extramural venous invasion (EMV-) (**Figure 1A**). Contrast-enhanced CT scans of the chest, abdomen, and pelvis revealed no distant metastasis, and the thymus was unremarkable. Pathological evaluation confirmed mucinous adenocarcinoma. The final diagnosis was locally advanced rectal cancer (LARC), mucinous adenocarcinoma, staged as cT4aN2aM0.

**Table 1:** Laboratory data before and after NAT.

Blood cell counts or chemistry	Before NAT	After NAT	Normal Range
WBC	5.4	5.77	3.50-9.50 (10^12/L)
RBC	4.61	4.69	4.3-5.8 (10^12/L)
HGB	89	93	130-175 (g/L)
CEA	24.23	3.42	0.00-5.00 (ng/ml)
CA199	1455.31	116.31	0.00-37.00 (U/ml)
CK	205	926	50-310 (U/L)
CK-MB	15	95	0-25 (U/L)
LDH	158	538	120-250 (U/L)
AST	15	74	15-40 (U/L)
ALT	8	58	9-50 (U/L)
CRP	6	0.8	0-6.0 (mg/L)

NAT, neoadjuvant therapy; WBC, white blood cell; RBC, red blood cell; HGB, hemoglobin; CEA, carcinoembryonic antigen; CA199, Carbohydrate antigen199; CK, creatine kinase; CK-MB, creatine kinase; MB form; LDH, lactate dehydrogenase; AST, alanine aminotransferase; ALT, alanine aminotransferase; CRP, c-reactive protein.

Following the diagnosis of LARC, the patient underwent NAT with a combination of CAPOX (oxaliplatin 130 mg/m² intravenously on day 1 and capecitabine 1000 mg/m² orally twice daily from day 1 to 14, every 3 weeks) and tislelizumab (200 mg intravenously on day 1, every 3 weeks). The patient developed right-sided ptosis on day 25 of NAT, demonstrating characteristic diurnal fluctuation (mild morning symptoms worsening by evening). Three days later (day 28), evaluation at a local hospital revealed elevated transaminases (ALT and AST; the exact values are unavailable), prompting hepatoprotective therapy with intravenous magnesium isoglycyrrhizinate and glutathione. NAT was discontinued at this time, though no neurological intervention was initiated.

Neurological progression ensued, with left-sided ptosis and cervical weakness emerging by day 32. By day 40, progressive limb weakness developed, culminating in severe functional impairment by day 60 (including inability to perform activities of daily living and requirement of cervical orthosis). The patient presented to our institution on day 62 post-NAT initiation, with no interim care received between discharge from the initial local hospital and this admission.

**Figure 2:**
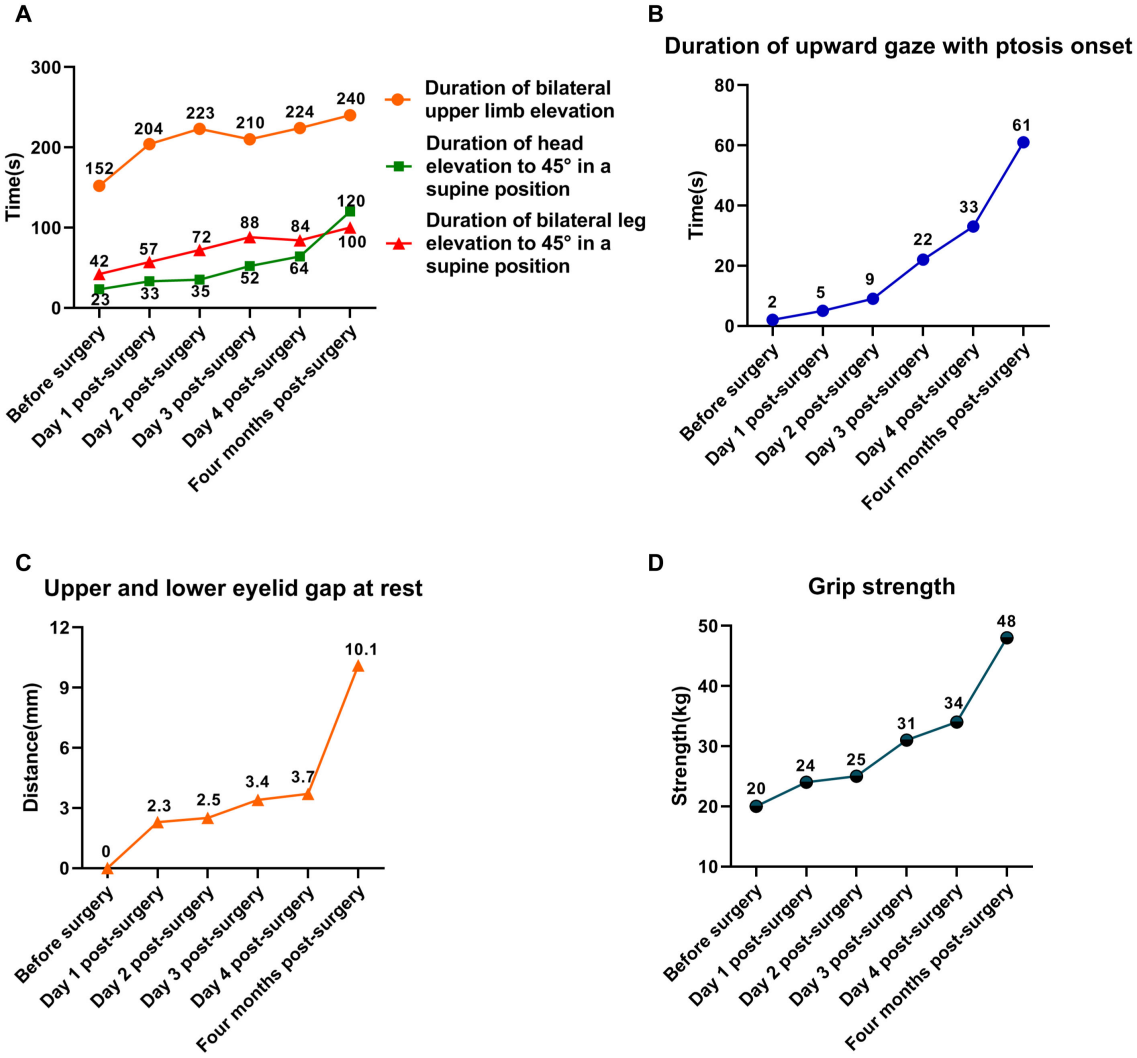
Trends in changes of muscle strength in the patient. **(A)** Trends in changes of limb and neck muscle strength. **(B)** Trends in changes of limb and neck muscle strength. **(C)** Trends in changes of upper and lower eyelid gap at rest. **(D)** Trends in changes of grip strength.

On physical examination, the patient exhibited reduced muscle strength in both the limbs and neck, along with bilateral ptosis (**Figure 2**). Ptosis developed after 2 seconds of upward gaze, with a resting upper and lower eyelid gap measuring 0 mm. In a seated position, he could sustain bilateral upper limb elevation at 90° for 152 seconds. In the supine position, he could maintain head elevation to 45° for 23 seconds and leg elevation to 45° for 42 seconds. Grip strength was recorded at 20 kg.

Laboratory tests (**Table 1**) showed a significant reduction in tumor markers compared to previous levels, along with an increase in muscle injury-related enzymes. Pelvic MRI and contrast-enhanced CT scans of the chest, abdomen, and pelvis revealed a reduction in the rectal tumor, now staged as T3N0M0 (**Figure 1B**), demonstrating significant tumor regression compared to prior imaging. No additional tumors, infections, thymic abnormalities, or muscle pathology were observed. The patient had no history of exposure to myotoxic agents. After excluding other causes, the patient's MG was suspected to be a paraneoplastic syndrome (PNS) secondary to rectal cancer.

The patient subsequently underwent laparoscopic radical resection of rectal cancer with a temporary ileostomy. On postoperative day 1, a marked improvement in muscle strength was observed (**Figure 2**). Ptosis occurred only after 5 seconds of upward gaze, and at rest, the upper and lower eyelid gap was 2 mm. Seated, the patient was able to sustain bilateral upper limb elevation at 90°for 204 seconds. In the supine position, he sustained head elevation to 45°for 33 seconds and leg elevation to 45° for 57 seconds. Grip strength was measured at 24 kg.

By postoperative day 4, the patient had made further gains in muscle strength and was discharged in stable condition, able to ambulate independently (**Figure 2**). Ptosis developed after 32 seconds of upward gaze, with an eyelid gap of 4 mm at rest. While seated, the patient maintained bilateral upper limb elevation at 90° for 221 seconds. In the supine position, he sustained head elevation to 45°for 64 seconds and leg elevation to 45°for 116 seconds. Grip strength had improved to 34 kg.

Postoperative pathological analysis confirmed an R0 resection, with cancer cells infiltrating the fibrofatty tissue beyond the muscularis propria. Both upper and lower resection margins were free of tumor involvement, and all 19 lymph nodes examined were negative for metastasis. The patient was advised to finish adjuvant chemotherapy. After two cycles of CAPOX, the patient refused to continue. Four months postoperatively, the patient returned for ileostomy closure. By this time, symptoms of MG had fully resolved, with muscle strength completely restored to normal levels (**Figure 2**).

## Discussion

This report presents a case of MG that developed during neoadjuvant chemotherapy combined with ICIs in a patient with LARC. Despite a five-week cessation of neoadjuvant therapy, the patient’s MG symptoms did not improve and showed signs of worsening, raising the suspicion of a PNS. Following surgical resection of the rectal tumor, the patient's MG symptoms began to improve on postoperative day 1, with significant recovery by postoperative day 5. Four months later, during ileostomy closure, the MG symptoms had fully resolved. The complete resolution of MG following radical tumor resection further supports the possibility of a diagnosis of a paraneoplastic etiology.

While MG is commonly associated with thymoma (18, 19), it can also arise as a paraneoplastic syndrome in other malignancies, including lymphoma, breast cancer, and lung cancer (12–17). The diagnosis of pMG is primarily based on a combination of oncological history, neurological manifestations, tumor response to treatment, and the presence of specific serum antibodies (20). In this case, a probable diagnosis of pMG was established based on the patient's oncological history, clinical manifestations, and treatment response. The temporal association between tumor resection and neurological improvement provides compelling clinical support for pMG. However, diagnostic limitations remain, including the lack of autoantibody testing (anti-AChR/MuSK) and confirmatory muscle biopsy, which precludes definitive exclusion of immune-mediated myositis. These gaps reduce the certainty of the pMG diagnosis and underscore the need for future prospective studies incorporating comprehensive serological and histological assessments.

Emerging evidence suggests molecular mimicry between tumor-associated antigens and nicotinic acetylcholine receptors (AChRs) at the neuromuscular junction may drive paraneoplastic MG (pMG) pathogenesis (21–23). Tumor-expressed abnormal antigens are misidentified by the immune system as acetylcholine receptors, thereby activating autoreactive T cells and assisting B cells in secreting anti-AChR antibodies. These antibodies directly block the function of AChR, leading to a significant reduction in the density of AChR at the postsynaptic membrane, disrupting synaptic transmission and ultimately resulting in myasthenia gravis (18, 23). While immune checkpoint inhibitors enhance antitumor immunity, they may break peripheral immune tolerance, activating cross-reactive autoimmune responses against the neuromuscular junction's acetylcholine receptors, thereby contributing to the development and progression of pMG (24). After the patient underwent surgical treatment, the continuous release of cross-reactive antigens was eliminated, interrupting the malignant cycle of the autoimmune response. This allowed for gradual restoration of acetylcholine receptor synthesis and synaptic structure, leading to the rapid alleviation of myasthenia gravis symptoms (11, 25–27). However, the exploration of this mechanism is still in its early stages, and further high-quality research is needed to investigate the underlying mechanisms more deeply.

Studies have shown that when ICIs are administered to tumors frequently associated with paraneoplastic syndromes, the incidence of PNS increases significantly. The underlying mechanism may be related to ICIs stimulating the body to enhance immune responses against potential neural antigens expressed in tumors (28). Yshii et al. (29) confirmed through their research that in mouse models with pre-existing subclinical paraneoplastic autoimmunity, ICIs can block CTLA-4 inhibitory signals, abrogate the suppression of pre-sensitized CD8^+^ cytotoxic T lymphocytes, enabling them to break through immune tolerance and further attack central nervous system cells expressing the same antigens, ultimately leading to the progression of subclinical lesions to clinical paraneoplastic syndromes.

In recent years, immunotherapy has become a pivotal strategy in cancer treatment (30, 31). While ICIs have demonstrated promising efficacy, they are also associated with a spectrum of immune-related adverse events (irAEs), with neurological irAEs occurring in approximately 1-5% of patients (10, 32). MG, as a neurological irAE, typically improves with discontinuation of the offending immunotherapy (33). However, in this case, the patient’s symptoms persisted and worsened after cessation of treatment, suggesting that this was not a typical irAE. Growing evidence indicates that ICIs can induce or exacerbate paraneoplastic neurological syndromes (34–36). In this patient, tumor resection likely led to antigen removal, resulting in a decrease in autoantibody levels and subsequent improvement in MG symptoms. This case likely represents pMG triggered by immunotherapy.

The current management of pMG typically includes corticosteroids, intravenous immunoglobulin, plasmapheresis, and immunosuppressants (37). Immunosuppressants may elevate the risk of anastomotic fistula by inhibiting fibroblast proliferation, blocking angiogenesis, and slowing the progression of neointimal hyperplasia (38–40). Currently, a significant proportion of rectal cancer patients undergo neoadjuvant chemoradiotherapy prior to surgery (2), and such patients exhibit a further increased risk of anastomotic fistula. When a patient’s myasthenic symptoms fail to resolve and instead exacerbate after discontinuing ICIs, a diagnosis of suspected pMG should be considered. Under such circumstances, radical surgical resection of the tumor may represent an effective therapeutic approach, as it not only achieves radical tumor eradication but also potentially ameliorates myasthenic symptoms. Moreover, given that the patient did not receive immunosuppressant therapy preoperatively, the risk of anastomotic fistula is relatively low. In our case, the patient’s limb muscle strength recovered more rapidly than the muscle strength in the face and neck after surgical intervention. This disparity may be attributed to the varying severity of MG symptoms across different regions during initial onset. However, as this observation is drawn from a single case, further research is necessary to determine whether this pattern is consistent in other patients.

In this case, the marked temporal correlation between tumor resection and resolution of myasthenia gravis symptoms strongly supports the possibility of a diagnosis of pMG. These findings offer novel insights into the management of MG in colorectal cancer, suggesting that surgical resection may represent an effective therapeutic strategy for patients developing MG symptoms during neoadjuvant therapy for LARC potentially avoiding complications associated with immunosuppressive therapies. For non-resectable disease, conventional MG management remains indicated.

## Data Availability

The raw data supporting the conclusions of this article will be made available by the authors, without undue reservation.
